# Rapid Microsatellite Isolation from a Butterfly by *De Novo* Transcriptome Sequencing: Performance and a Comparison with AFLP-Derived Distances

**DOI:** 10.1371/journal.pone.0011212

**Published:** 2010-06-18

**Authors:** Alexander S. Mikheyev, Tanya Vo, Brian Wee, Michael C. Singer, Camille Parmesan

**Affiliations:** 1 Okinawa Institute of Science and Technology, Onna, Japan; 2 National Ecological Observatory Network, Incorporated, Washington, District of Columbia, United States of America; 3 University of Texas, Section of Integrative Biology, Austin, Texas, United States of America; Department of Animal Ecology, Lund University, Sweden

## Abstract

**Background:**

The isolation of microsatellite markers remains laborious and expensive. For some taxa, such as Lepidoptera, development of microsatellite markers has been particularly difficult, as many markers appear to be located in repetitive DNA and have nearly identical flanking regions. We attempted to circumvent this problem by bioinformatic mining of microsatellite sequences from a *de novo*-sequenced transcriptome of a butterfly (*Euphydryas editha*).

**Principal Findings:**

By searching the assembled sequence data for perfect microsatellite repeats we found 10 polymorphic loci. Although, like many expressed sequence tag-derived microsatellites, our markers show strong deviations from Hardy-Weinberg equilibrium in many populations, and, in some cases, a high incidence of null alleles, we show that they nonetheless provide measures of population differentiation consistent with those obtained by amplified fragment length polymorphism analysis. Estimates of pairwise population differentiation between 23 populations were concordant between microsatellite-derived data and AFLP analysis of the same samples (r = 0.71, p<0.00001, 425 individuals from 23 populations).

**Significance:**

*De novo* transcriptional sequencing appears to be a rapid and cost-effective tool for developing microsatellite markers for difficult genomes.

## Introduction

Many types of genetic analysis take advantage of microsatellite markers, which are highly polymorphic loci of simple sequence repeats located through the genome. For example, microsatellite analysis is useful in studies of paternity, population structure and history, as well as to make conservation decisions for the management of endangered species [1], [2].

Given the broad-scale utility of these markers, a large number of approaches have been developed for their isolation from genomic DNA [3]. These approaches typically involve some form of microsatellite enrichment, followed by time consuming and costly brute force sequencing. Aside for the labor and cost associated with traditional approaches, the microsatellite enrichment step sometimes fails. For example, for reasons not fully understood, isolation of microsatellites from Lepidopteran genomes is extremely difficult [4]–[6]. This problem is not confined to Lepidoptera, affecting bivalve mollusks [7], mosquitoes [8], mites [9], ticks [8], nematodes [10], [11] and birds [12], [13].

The increase in publicly available EST data for many species has made bioinformatic isolation of microsatellite markers increasingly commonplace (*e.g.*, [14]–[17]). However, microsatellites isolated from EST libraries differ from those typically found in regions of the genome unassociated with genes. Gene-associated microsatellites are physically linked to particular alleles of a gene, and may hitchhike if the gene is under selection. Microsatellite variation in untranslated regions of transcribed DNA may affect the rates of gene expression or translation, and thus may be under selection. Indeed, EST-derived microsatellites almost universally show strong deviations from Hardy-Weinberg equilibrium. However, the relatively few studies that compare the performance of EST-derived microsatellites with that of other genotyping techniques have generally found comparable results [18]–[22]. Here we used the Roche 454 Titanium platform for transcriptional sequencing of Edith's checkerspot butterfly (*Euphydryas editha*), in order to rapidly isolate polymorphic microsatellite loci for a conservation genetics study. We then compared the estimates of population differentiation and biogeographic structure obtained by this approach with those from AFLP genotyping of the same set of populations [23].

## Materials and Methods

### Microsatellite identification

RNA was extracted from a larva, a pupa and an adult *E. editha*. RNA extraction, normalized library preparation, sequencing and assembly using the Roche Newbler assembler was performed by the University of Illinois W.M. Keck Center for Comparative and Functional Genomics using protocols and reagents supplied by Roche. The assembled data were then queried for the presence of microsatellites using a simple python script using all possible sequences combinations of di-, tri- and tetra-nucleotide repeats, with at least eight perfect repeats. Primers for microsatellite-containing sequences were designed using Primer3 [24] and tested for amplification and polymorphism.

### Microsatellite amplification and polymorphism testing

Microsatellite loci were tested for amplification and polymorphism in 10 µl PCR mixes containing 1 ng genomic DNA, 10 mg BSA, 10 pmol primers, 6.7 nmol of ChromaTide® Rhodamine Green™-5-dUTP (Molecular Probes, presently discontinued) and 5 µl AmpliTaq Gold® PCR Master Mix (Applied Biosystems). The temperature cycling conditions were as follows: 7 min at 94°C, then 35 cycles of 10 sec at 94°C, 1.5 minutes at 60°C and 2 minutes at 68°C. The reaction was terminated with a final incubation of 30 minutes at 72°C. 1 µl of each reaction was then analyzed using an ABI3100 DNA sequencer. For genotyping each well had 0.1 µl LIZ labeled GeneScan 500 size standard (Applied Biosystems) and enough deionized formamide for a total volume of 10 µl. Alleles were scored using GeneMarker.

### Quality control

Deviations from Hardy-Weinberg equilibrium were assessed using GenAlEx [25]. Many individuals in the present study were previously genotyped by Wee [26] using AFLP markers. Thus, we were able to assess concordance between results of the two studies by comparing Fst matrixes generated by the two techniques. We computed Fst distances for 23 populations (425 individuals) used in Arlequin (v.3) [27], and compared them to the Fst matrix from Wee [26] using a Mantel test with 10,000 bootstrap replicates. We also screened an additional 406 individuals from 48 more populations for polymorphism analysis (Table S1).

## Results

After quality filtering, the 454 run generated 864,056 reads, totaling 245,064,986 bases, which were assembled into 14,244 contigs with a threshold of 200 bp overlap and 95% identity. 49,937 singleton reads remained unassembled and were not included in the subsequent analysis, although if needed, they may be used for microsatellite mining. The assembled contigs contained 92 microsatellite loci, 72 of which were selected for microsatellite development. Of these, 36 loci amplified successfully and appeared polymorphic (see Table S2). Following the initial screening performed of eight individuals, we developed four multiplex PCR cocktails containing a total 10 polymorphic loci for large-scale genotyping (Table S1). Sequences for the other loci are available from the authors upon request. The reaction conditions were as above, but without fluorescently labeled dUTPs in reactions 1 and 2, and with primer concentrations as noted in Table S2. The 10 loci are deposited in GenBank under accession numbers GU997598-GU997607.

**Figure 1:**
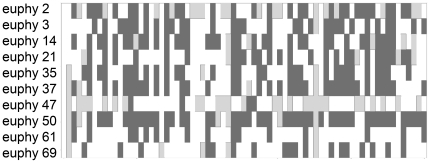
Hardy-Weinberg equilibrium statistics. Significant deviations from Hardy-Weinberg (chi-squared test, p<0.05) are indicated in dark grey. Loci monomorphic in that population are shown in light grey. Every population is represented by a column, with each row corresponding to a microsatellite locus. The order of the populations is the same as in Table S1 (alphabetical).

The markers show significant deviations from Hardy-Weinberg equilibrium in the many of the populations (Figure 1). The difference between observed and expected heterozygosities was positively correlated with the number of failed amplification for each locus, suggesting that null alleles may in part be responsible for driving this difference (r_s_ = 0.81, n = 10, p = 0.0042). However, estimates of pairwise population differentiation were concordant between microsatellite-derived data and an earlier AFLP analysis of the same samples by Wee [26] (r = 0.71, n = 23, p<0.00001).

Raw microsatellite data generated in this study have been deposited in the Dryad database (www.datadryad.org) under accession number 1540.

## Discussion

Microsatellite isolation from lepidopteran genomes has been difficult, possibly because microsatellite loci appear to be rare, and may have very similar flanking regions [6], which makes the design of primers problematic. We hypothesized that microsatellite loci isolated from non-translated transcripts may be less likely to exist as duplicate copies, and thus be more amenable to marker development. This has made microsatellite isolation relatively straightforward in our case. Given the decrease in next-generation sequencing costs, transcriptional re-sequencing will be a faster and cheaper way to isolate microsatellites, compared with traditional enrichment techniques. We were able to complete microsatellite development and screening in about three months of part time work by a single technician. Our actual cost of library construction and sequencing, was about US$15,000, is comparable to that charged by private companies for microsatellite enrichment [3]. Since then, the actual cost of library construction and next generation sequencing has dropped by at least 50%, and is decreasing further.

In this and several other studies, microsatellites derived from transcribed sequence data significantly depart from Hardy-Weinberg equilibrium (Figure 1) [14]–[17]. This could be due to selection on polymorphisms in untranslated gene regions where these microsatellites typically reside, or to non-neutral dynamics of the genes to which they are physically linked. In our study, percent reaction failure explained most of the variance in the differences between observed and expected heterozygosities (Table 1). Therefore, at least in our case, Hardy-Weinberg disequilibrium may be partially due to insufficient optimization of PCR conditions and allele dropout. Whether or not higher levels of null alleles are common in EST-derived microsatellites is not clear, since these data are not routinely reported with such studies. We strongly recommend further optimization of the reaction conditions for the loci presented here, especially since the manufacture of fluorescent dUTPs used in this study has been discontinued.

**Table 1:** Primers used for large-scale genotyping.

PCR #	Locus	Primers sequence	Primer amount (pmol)	Label	Repeat Motif	Range (bp)	Allele Count	H_o_	H_e_	Percent missing
1	euphy 2	tgatgataacgagcgggaag	0.5	5′TAM	CAG	144–191	20	0.42	0.72	0.60%
		cggtaccgctacgtgactact								
	euphy 3	gctgtaatttggtaaggggttg	0.5	5′ HEX	ATC	121–171	18	0.52	0.83	0.84%
		tacgttcagtgatggacatgc								
	euphy 21	acgcaaggtgctccacttat	0.5	5′ HEX	CAA	220–239	9	0.18	0.24	1.32%
		ttgctacgctaacagcatcg								
	euphy 69	ctcctccgcaccaacaagta	1	5′ FAM	GTT	72–103	13	0.17	0.39	3.59%
		aaacgtctacgttagaaggtatgt								
2	euphy 14	tgactgaacacacggacgat	0.5	5′ TAM	TACA	99–170	32	0.15	0.68	14.0%
		tccatcatgctttaagtgagga								
	euphy 61	aaagcgtgcttacattacatgg	0.5	5′ TAM	AC	186–246	42	0.44	0.87	12.9%
		tcccgtttaacataatctgtgg								
3	euphy 35	atagaaataaacatgcggccata	10	dUTP	TG	267–335	56	0.33	0.96	13.1%
		cagatgtacaagaggctgcctta								
	euphy 50	atgcgatttcatgccacata	10	dUTP	CA, A	135–176	28	0.22	0.85	22.5%
		ccatcctgacatgtgaaacg								
4	euphy 37	tgcaagacttgaaatatggttatca	10	dUTP	C, CA	130–182	21	0.41	0.80	2.28%
		gtccattggaaggatcagga								
	euphy 47	cacgtgagcattccagtttg	10	dUTP	AT	172–335	34	0.44	0.87	5.99%
		tcggcgtaacggtttaaatg								

Summary statistics are based on a survey of 835 individuals from 72 populations (Table 1). Even and odd numbered reactions were pooled and analyzed together in the same sequencer run. The percentages of missing were significantly different among the PCR mixes, being significantly higher in reactions 2 and 3 (F_3,6_ = 15.4, p = 0.0038).

In principle, deviations from Hardy-Weinberg can create substantial biases [28], limiting the utility of such markers. The extent to which these issues may affect analysis with EST-derived microsatellites is presently unclear, but should be carefully investigated by future studies. Ideally, studies isolating microsatellites from ESTs should verify their performance by comparing results with another genotyping method, as we have done with AFLPs. Likewise, it would be useful to present an analysis of null allele presence.

## Supporting Information

Table S1 Sample sizes and locations of the populations used for polymorphism screening.
(0.10 MB DOC)

Table S2 This file lists all the primers tested in the study, and the results of polymorphism testing based on a small sample of 8 individuals. Loci used for further analysis are highlighted in gray.
(0.12 MB DOC)
