# Supplementary material for: Rapid Microsatellite Isolation from a Butterfly by De Novo Transcriptome Sequencing: Performance and a Comparison with AFLP-Derived Distances
Source: PLoS One. 2010 Jun 18;5(6):e11212. doi: 10.1371/journal.pone.0011212 (PMC2887849; doi:10.1371/journal.pone.0011212)
Supplement: Table S1 — Sample sizes and locations of the populations used for polymorphism screening. (0.10 MB DOC) [file pone.0011212.s001.doc]

Table S1. Sample sizes and locations of the populations used for polymorphism screening.

| **Population name** | **Latitude** | **Longitude** | **Microsatellite**  **sample size** | **AFLP**  **sample size** |
| --- | --- | --- | --- | --- |
| Agua Fria | 37.50 | -120.17 | 6 | 0 |
| Almont Summit Colorado | 38.63 | -106.90 | 6 | 0 |
| Ash canyon | 41.83 | -122.60 | 15 | 0 |
| Balch Camp | 36.91 | -119.12 | 2 | 0 |
| Barbara Trail | 33.54 | -116.70 | 4 | 0 |
| Big Baldy | 36.69 | -118.87 | 11 | 0 |
| Big Meadow | 35.88 | -118.35 | 13 | 14 |
| Bircham Flat Road | 38.45 | -119.45 | 20 | 10 |
| Buck Rock | 36.73 | 118.86 | 10 | 10 |
| California Hot Springs | 35.88 | -118.68 | 4 | 0 |
| Cardwell Hill Rd | 44.61 | -123.35 | 15 | 0 |
| Chrome Ridge | 42.46 | -123.73 | 12 | 0 |
| Colony Meadows | 36.62 | -118.60 | 13 | 12 |
| Crawford Creek | 41.17 | -123.10 | 15 | 0 |
| Dana Meadows | 37.90 | -119.26 | 17 | 14 |
| Del Puerto Canyon | 37.44 | -121.49 | 13 | 13 |
| Dubakella Mtn | 40.38 | -123.14 | 15 | 0 |
| Dulzura | 32.63 | -116.63 | 6 | 0 |
| Ebbett's Pass | 38.55 | -119.82 | 15 | 10 |
| Franklin Point | 40.05 | -122.69 | 6 | 0 |
| Frenchman Lake | 39.95 | -120.21 | 17 | 15 |
| Gardisky Lake | 37.97 | -119.25 | 5 | 0 |
| Glen Alpine | 38.88 | -120.11 | 4 | 0 |
| Gold Lake | 39.67 | -120.67 | 12 | 11 |
| Indian Flat | 37.66 | -119.84 | 7 | 0 |
| Iowa Hill | 39.10 | -120.93 | 4 | 0 |
| Jacumba | 32.62 | -116.20 | 3 | 0 |
| Kingston Canyon | 39.23 | -117.15 | 3 | 0 |
| Knoxville Road | 38.47 | -122.19 | 14 | 0 |
| Leek Springs | 38.64 | -120.24 | 10 | 11 |
| Look Out Point | 36.74 | -118.86 | 5 | 0 |
| Lower Buck Rock | 36.73 | -118.86 | 6 | 0 |
| Mammoth crest | 37.60 | -119.03 | 1 | 0 |
| Marron Valley | 32.57 | -116.75 | 27 | 0 |
| McGee | 37.31 | -118.61 | 42 | 39 |
| Mill Canyon | 38.47 | -119.51 | 10 | 0 |
| Monache Meadows | 36.20 | -118.18 | 4 | 0 |
| Morgan Hill | 37.18 | -121.70 | 19 | 19 |
| Mount Dana | 37.90 | -119.22 | 10 | 10 |
| Mud Creek | 39.90 | -121.70 | 7 | 0 |
| Obrien Bog | 42.07 | -123.72 | 14 | 0 |
| Otay Mountain | 32.58 | -116.89 | 3 | 0 |
| Parker Pass | 37.83 | -119.20 | 12 | 10 |
| Pequop Mountain | 41.05 | -114.59 | 5 | 0 |
| Piute | 35.46 | -118.38 | 11 | 0 |
| Powerhouse Road | 37.14 | -119.52 | 8 | 0 |
| Pozo | 35.30 | -120.48 | 24 | 0 |
| Pratt's | 33.47 | -116.64 | 17 | 0 |
| Rabbit Meadow | 36.71 | -118.87 | 8 | 0 |
| Ramona | 33.08 | -116.91 | 1 | 0 |
| Road to Emma Lake | 38.31 | -119.45 | 6 | 0 |
| Rowell Meadows | 36.72 | -118.81 | 12 | 10 |
| Ruth Reservoir | 40.33 | -123.40 | 15 | 0 |
| Saddlebag Lake | 37.98 | -119.29 | 11 | 0 |
| Saddlebag Ridge | 37.97 | -119.26 | 13 | 10 |
| Schneider Meadows | 39.08 | -119.83 | 13 | 13 |
| Silverado Ranch | 33.73 | -117.64 | 16 | 16 |
| Skinner | 33.59 | -117.06 | 6 | 0 |
| Snow Mtn | 39.38 | -122.77 | 10 | 0 |
| Sonora Junction | 38.37 | -119.48 | 51 | 34 |
| Spillway Lake | 37.83 | -119.22 | 11 | 0 |
| T Junction distal | 36.71 | -118.83 | 50 | 32 |
| Tahoe Meadows | 39.31 | -119.89 | 5 | 0 |
| Tamarack Ridge | 37.21 | -119.19 | 17 | 17 |
| Timber Gap | 36.48 | -118.57 | 12 | 12 |
| Toiyabe | 39.26 | -117.73 | 8 | 0 |
| Tuolumne Meadows | 37.87 | -119.35 | 12 | 11 |
| Walker Pass | 35.65 | -118.04 | 9 | 0 |
| Warner Mt | 41.20 | -120.16 | 5 | 0 |
| Wild Horse Springs | 34.20 | -116.77 | 9 | 0 |
| Yucca Point | 36.82 | -118.89 | 9 | 0 |
