# Supplementary material for: Rapid Microsatellite Isolation from a Butterfly by De Novo Transcriptome Sequencing: Performance and a Comparison with AFLP-Derived Distances
Source: PLoS One. 2010 Jun 18;5(6):e11212. doi: 10.1371/journal.pone.0011212 (PMC2887849; doi:10.1371/journal.pone.0011212)
Supplement: Table S2 — This file lists all the primers tested in the study, and the results of polymorphism testing based on a small sample of 8 individuals. Loci used for further analysis are highlighted in gray. (0.12 MB DOC) [file pone.0011212.s002.doc]

Table S2. This file lists all the primers tested in the study, and the results of polymorphism testing based on a small sample of 8 individuals. Loci used for further analysis are highlighted in gray.

| **Primer** | **Motif** | **Forward Primer** | **Reverse Primer** | **Size Range** |
| --- | --- | --- | --- | --- |
| euphy01 | (GCA)12 | CCCCACTCCAGTACTCTTGC | CACGGTGACTTGTGTGATCC | no product |
| euphy02 | (CAG)12 | TGATGATAACGAGCGGGAAG | CGGTACCGCTACGTGACTACT | 156-168 |
| euphy03 | (ATC)8 | GCTGTAATTTGGTAAGGGGTTG | TACGTTCAGTGATGGACATGC | 121-153 |
| euphy04 | TCA(14) | CGGTGGTAGTCATGGAGTTTC | CTCTGGTCGATGGCCTATGT | 140-180 |
| euphy05 | (GTT)12 | GAAAACGTCTACGTTAGAAGGTATGTT | CACGGAAGAAAACGCTTAGG | 192-199 |
| euphy06 | GTT16 | CCTCTGAAGAGCAGCCTGAA | GAACCAAGGTCAACCAGCAC | no product |
| euphy07 | GAT14 | GGACATCCAATGGCTGAAGT | GCAGTAATAATCTCGTGCTGCTT | 137-138 |
| euphy08 | GAT9 | ATTCGACCCAAGCAATGAAT | CGTCTTCATACGGCTGTTCC | no product |
| euphy09 | TGA11 | CGTAAGATTCGGGTCAATGG | GGATTTCTCGTGTCCCTCAA | no product |
| euphy10 | TGC10 | GGTCAGCACACTTGGAACAG | AAGTACTTCCCCACCTGACG | no product |
| euphy11 | ATG14 | CCATCCAAAGCGTCAAAACT | AAGCGATTACGGGAGCATC | 235-265 |
| euphy12 | TCA9 | GCAGTGGTATCAACGCAGAG | TACGTACCACCAGCAGTGGA | no product |
| euphy13 | ACAT8 | TTTGTGTTATAACCTGTCCGAAA | CATGCTGTTAAAGGAATTTTCAA | no product |
| euphy14 | TACA12 | TGACTGAACACACGGACGAT | TCCATCATGCTTTAAGTGAGGA | 113-124 |
| euphy15 | TACA9 | AAAGGTGGAAACGGTGACAT | TAAACGCTAGCCATCACGTC | no product |
| euphy16 | TAAA9 | TGGTCTTGTTTATTGCTGTAACG | ACGTATCGCTTGCTCGCTTA | no product |
| euphy17 | CTG9 | TATTGTGATGCCACGCTCA | GCAGTGGTATCAACGCAGAG | no product |
| euphy18 | TCA12 | CTGTTTCAACTTCGGTTTCTCT | CCTACGTTCAGCAGTGGACA | no product |
| euphy19 | TAA10 | TGGAGGAGTTTCATGAATTTGA | GTGGACTCAACACCCCACAT | no product |
| euphy20 | TGA31 | CGTACACGCGCTAGAAGGAG | ACAAATCCAACGACACCTCA | bad amp |
| euphy21 | (CAA)8 | ACGCAAGGTGCTCCACTTAT | TTGCTACGCTAACAGCATCG | 226-236 |
| euphy22 | (TA)10(CTATA)4 | AAACTGCAGCCAGATACTCCT | CATCGCTAACACAGCGATTT | 158-206 |
| euphy23 | (TA)9 | TACGCAATTTGGAATCGTCA | ATCCATGCCAGTTGTTGAAA | no product |
| euphy24 | (TG)14 | TATCATCAAACCTGGCATCG | GCTGTGACGTTCGTGGTTTA | 233-250 |
| euphy25 | (TA)9 | TGAAATCGGCCACTTCATTT | TATGTCCAGCAGTGGTCAGC | no product |
| euphy26 | (CAA)7 | CCCGAGACCAAGTCCAAGTA | CATCGTCGCCTTCAAATTCT | no product |
| euphy27 | (ATC)9 | GGATTTCTCGTGTCCCTCAA | ATGCACTTCGAAACGCTACC | 235-255 |
| euphy28 | (TG)12 | AGGGCATTGAAAGCACAAGT | TGCATTCAGCTTCATGTCTAGG | 207-229 |
| euphy29 | (AC)8 | TCAGAAGATAGGTCAATATCCCTGT | CGTGTTGACCGTACATGACA | no product |
| euphy30 | (TA)5 - (TTG)8 - (TGT)7 - (GTT)5 | GCACCATTTCAAACAGCACA | CAGGGCTTCAACAACAACAG | 218-253 |
| euphy31 | (CAG)6 - (CAA)8 | CAAGAAGAAATTCAACGCTTACAA | TGCTGTTGATATTGATGGTAATTT | messy |
| euphy32 | (TG)9 | CATTGTACAAATTGAATTGGAATG | CGCATAAGACGCACGAAATA | messy |
| euphy33 | (GT)10 | ATCGATGGGTTGACAGAAGC | CCAGAAACAAATACGTTCATCG | no product |
| euphy34 | (A)8 - (TA)9 | CGTTCCCATCCGAGCTATTA | TGTACATCTTATATCTTTAAACGAGCA | no product |
| euphy35 | (TG)8 | ATAGAAATAAACATGCGGCCATA | CAGATGTACAAGAGGCTGCCTTA | 271-311 |
| euphy36 | (TA)8 | TTCTCATGCCATTTGTCTGTT | TCCCACTGGACTGTGAGAAA | 249-257 |
| euphy37 | ( C)10 - (CA)9 | TGCAAGACTTGAAATATGGTTATCA | GTCCATTGGAAGGATCAGGA | 161-176 |
| euphy38 | (TA)8 - (TA)5 | GCATTATTTCGAGGCCCTAAC | CACAAATGGACGCATTAGCA | 262-287 |
| euphy39 | (AG)8 | CTTGTGGAGTACGCTGGTGA | AAATCTCTGCACCTCTGTTGC | no product |
| euphy40 | (TG)4 - (AT)11(TG)5 | TTGAATCGGATGTCACCAAA | TGTATACATACAGAGGAATCGATGAA | no product |
| euphy41 | (TG)4 - (GT)11 | CGACTGCGACAGAAGGAGAG | GCAATCGTTCCCATCCTAAA | 159-173 |
| euphy42 | (TA)8 - (T)8 | CAAGATGCCGGTAATTCAGA | TGAAACAATATTCATACATAAACTTCG | 255-264 |
| euphy43 | (ATG)12 | CCTACGTTCAGCAGTGGACA | TGTTTCAACTTCGGTTTCTCAA | 191-216 |
| euphy44 | (GT)9 - (TA)4 | GGGACCGATGtCtGTTtCtC | TCCAAAGTGATaTAAACTCATGTGaTa | 233-292 |
| euphy45 | (AC)8 | GaCGAaGCAaCTGACAGCAC | ACCATCCAATGACCTTGAGC | 185-227 |
| euphy46 | (AT)9 | AAaCGCTATACAATTTGGATCAAT | TAGAGCGAtTCCCAGAcCAT | 214-240 |
| euphy46A_RVS | (AT)9 |  | GAGAAACTGAATTTTTTG |  |
| euphy47 | (AT)8 - (AT)4 | CACGTGAGCATTCCAGTTTG | TCGGCGTAACGGtTTAAATG | 302-308 |
| euphy48 | (TATG)5 - (TA)9 | TTTGCATTTACGTtcTTCAaTTT | cCCAACAGAAAgGAGCTTGA | no product |
| euphy49 | (AT)9 | TTTAGCACATAGGTGATGAATCTCT | CCTTAAACCCGaGCAAGCTA | 159-165 |
| euphy50 | (CA)13 - (A)10 | aTGCGATTTCATGCCACAtA | CCATCCTGACATGTGAAACG | 135-176 |
| euphy51 | (AT)6 -T-(AC)5(TA)4(CA)5(TA)4(CA)8-TG-(TA)4 | TTCAAGAGCTGGTGCTTCTTC | CTGCATTGTcCAAAGGTCAC | 238-316 |
| euphy52 | (TATAG)4-G-(TA)10 | TTCTTCATGATATAGCTCCTACGG | GACATGAgGTCGGTATATGGaAA | no product |
| euphy53 | (AT)8 - (AT)4 | AGAGGAGCTTGGGAGGCTAC | AAGCTGTGACCCAGGGACTA | 201-205 |
| euphy54 | (TA)8 | TGTTATAGCGAACTGTGGCTAGG | TTGTAcCTAGCAATGCAATAAAGA | 203-251 |
| euphy55 | (AC)4-GA-(AC)13 | CCCAATGTTCCAGGTAATTGTT | TACAACGCGATTGTCGGTTT | 300-406 |
| euphy56 | (T)8 - (AT)10 | AACATAAACTAACATATACGCGACAA | AAGGCTCATCTTAAGTTGGTGTA | no product |
| euphy57 | (AT)10 | TTTGAGCCGAAGTTTATATGTATCT | CGTTGACCTGCGAAGTTTGT | no product |
| euphy58 | (AT)9 | TTTACAACATAATCGCCGTTC | TTTCTATGATTTATCCAAATGTAAAGC | no product |
| euphy59 | (A)14(AC)10 | TGTGCACACATGCAGAGAAC | GCGTGTGCAAGTGTGTGTAA | 212-236 |
| euphy60 | (AC)7(AT)9 - (AC)4 | TGTATACATACAGAGGAATCGATGAA | TTGAATCGGATGTCACCAAA | 198-220 |
| euphy61 | (AC)11 | AAAGCGTGCTTACATTACATGG | TCCCGTTTAACATAATCTGTGG | 202-258 |
| euphy61A_RVS | (AC)11 |  | GTGGTTCTAAGTGAGAGA |  |
| euphy62 | (AT)9(T)8 | AAGCGAAATCATACACATCTCG | AAGCGTGACTGACTAACGAACA | 224-274 |
| euphy63 | (AC)11(CACG)8-A-(ACGC)4 | TGAAATGAAATGATAATGAAATGGA | CGGCCAGCACTAAAGACATT | 173-181 |
| euphy64 | (AGT)8 | ACGTGACCGACGTGACATTA | GCAGAAGATTCGGTTATGTGG | no product |
| euphy65 | (AT)8 - (TA)4 | GTTAGCCTCTTTCGAGAGTGAA | TCCTACCTTCAAATACGCAATAC | 175-385 |
| euphy66 | (ATG)8 | AGAGCCACGAGTCGAGCTTA | AGGCGCGACTCAAGTGTAAC | 202-259 |
| euphy67 | (GT)8 | CGCAACCTCTCTCCGATCTA | GGCTAACACCTCGACATTGG | 158-165 |
| euphy68 | (AT)9 | TTTGATTCATCACCAACACAAA | GGCTTAAGCGATTCTAGCAACA | 247-266 |
| euphy69 | (GTT)12 | CTCCTCCGCACCAACAAGTA | AAACGTCTACGTTAGAAGGTATGT | 76-92 |
| euphy70 | (TG)4 - (GT)11 | GAGACGTTAGGTTCGATCCTTCT | GCAATCGTTCCCATCCTAAA | no product |
| euphy70A _RVS | (TG)4 - (GT)11 |  | CGTTCAGTGATGCAATCGTT | no product |
| euphy70B _RVS | (TG)4 - (GT)11 |  | ATGCAATCGTTCCCATCCTA | no product |
| euphy70C _RVS | (TG)4 - (GT)11 |  | ATCGGCCAACTCCAAATACA | no product |
| euphy70D _RVS | (TG)4 - (GT)11 |  | TTCAGTGATGCAATCGTTCC | no product |
| euphy71 | (AT)9 | GCTTAGGTTCAAACTaTCCgAAa | CCTTAAACCCGaGCAAGCTA | 106-112 |
| euphy71A_RVS | (AT)9 |  | TTCATCACCTATGTGCTAAACATC |  |
| euphy72 | (AC)7(AT)9 - (AC)4 | TGTATACATACAGAGGAATCGATGAA | CGTTATAGCCCACGTTGTCC | 266-296 |
